# Knockdown of the Inhibitor of Apoptosis BRUCE Sensitizes Resistant Breast Cancer Cells to Chemotherapeutic Agents

**DOI:** 10.4172/1948-5956.1000335

**Published:** 2015-04-15

**Authors:** Jason B Garrison, Chunmin Ge, Lixiao Che, Derek A Pullum, Guang Peng, Sohaib Khan, Nira Ben-Jonathan, Jiang Wang, Chunying Du

**Affiliations:** 1 Department of Cancer Biology, University of Cincinnati College of Medicine, Cincinnati, USA; 2 Department of Clinical Cancer Prevention, The University of Texas MD Anderson Cancer Center, Houston, USA; 3 Department of Pathology and Laboratory Medicine, University of Cincinnati College of Medicine, Cincinnati, USA

**Keywords:** DNA damage repair and signaling, Inhibitor of apoptosis protein (IAP), Cell death, Chemotherapy, Breast cancer cells, Breast tumors

## Abstract

**Background and objectives:**

Management of patients with breast cancer often fails because of inherent or acquired resistance to chemotherapy. BRUCE (BIR repeat containing ubiquitin-conjugating enzyme) is a member of the inhibitor of apoptosis protein (IAP) family. It has various cellular functions including suppression of apoptosis and promotion of cytokinesis. Furthermore, it pays a critical role in promotion of DNA damage repair and preservation of genome stability, a new function recently reported by our group. Although BRUCE is expressed in breast cancer cell lines, its expression in human primary breast tumors and its contribution to chemoresistance in breast cancers has not been explored. Chemotherapeutic drugs are used in the treatment of breast cancer patients. However, they are not effective to all patients and patients often develop resistance. Consequently we explored if BRUCE protein level, as judged by immunohistochemistry (IHC), is higher in primary breast tumors than normal breast tissue. We also examined if depletion of BRUCE, using a lentiviral shRNA approach, enhances cell sensitivity to multiple chemotherapeutic agents, including cisplatin, an agent that induces DNA damage by generating DNA cross-links, and taxol, a microtubule stabilizer and mitotic inhibitor. The reason for including these two chemotherapeutic agents in this study is that they hit two essential cellular processes of DNA repair and cytokinesis in which BRUCE plays critical roles.

**Results and methods:**

IHC analysis of BRUCE revealed significantly higher levels of BRUCE in primary breast tumors than normal breast tissue. Knockdown of BRUCE protein expression by lentiviral shRNA resulted in increased sensitivity to cisplatin in the resistant breast cancer MDB-MD-231 cell line. Moreover, depletion of BRUCE in this cell line achieved a more profound level of cell killing when coupled to low doses of cisplatin and taxol combined, rather than either drug used alone.

**Conclusions:**

Our data suggest that elevated protein levels of BRUCE in breast tumors may contribute to chemoresistance in breast cancer patients. In support of this suggestion, our data demonstrate that a reduction in BRUCE expression in breast cancer cell lines increases the toxicity of several chemotherapeutic agents. In all likelihood, the contribution of increased BRUCE levels to chemoresistance are likely due to its roles in suppression of apoptosis, promotion of cytokinesis and facilitation of DNA damage repair. These observations suggest that therapeutic suppression of BRUCE could improve chemosensitivity in chemo-resistant breast cancer patients. Therefore, future development of effective inhibitors of BRUCE could benefit patients with high BRUCE expression and chemoresistance.

## Introduction

Breast cancer is the most common malignancy among women in the United States with approximately 30% of patients developing metastatic disease [1,2]. Resistance to chemotherapy remains a significant obstacle in the treatment of breast cancer. Cisplatin and taxol are widely used for treating early-stage and metastatic breast cancer. Cisplatin is an alkylating-like compound that causes covalent DNA adducts leading to cell death, whereas taxol is a microtubule-stabilizing agent that arrests cells in mitosis leading to cell death [3–5]. These drugs are not effective in all patients, and the molecular basis underlying resistant vs sensitive tumors is not well understood [6]. Therefore, the identification of endogenous proteins associated with chemoresistance to cisplatin and taxol should enable the development of novel drugs for treating patients that do not respond to such treatment.

The BIR repeat containing ubiquitin-conjugating enzyme (BRUCE) is a giant multifunctional protein (528 kDa). It is a member of the inhibitor of apoptosis protein (IAP) family [7,8], which is defined by containing the baculoviral IAP-repeat (BIR) domain in all IAP proteins. BRUCE contains one BIR domain near its N-terminal region [9,10]. In addition, BRUCE also has a ubiquitin protein conjugating (UBC) [11] and a midbody ring-targeting domain (MTD) near its C-terminus [12]. Because of technical challenges associated with studying high molecular weight proteins, the biological functions of BRUCE are not completely understood. The IAP function of BRUCE is mediated by the binding of its BIR domain to caspases-3, 6, 7, and 9 in order to inhibit caspases and suppress apoptosis [11,13]. In addition, the UBC domain of BRUCE has both ubiquitin conjugase (E2) and ligase (E3) functions in vitro. The E2/E3 function of BRUCE is believed to promote cell survival by degradation of pro-apoptotic proteins, including the mitochondrial proteins Smac/Diablo [11,14–16] and serine protease HTRA2/Omi [11,17], as well as cytoplasmic caspase-9 [14]. Furthermore, the MTD domain confers BRUCE a role in the promotion of cytokinesis. Specifically, BRUCE is localized to the midbody of a cell during cytokinesis and required for mitotic cell division. As a result, depletion of BRUCE by siRNA results in aborted cytokinesis and mitotic cell death [12]. In addition to inhibiting apoptosis and promoting cytokinesis, BRUCE plays an essential role in DNA damage repair and genome stability preservation recently reported by our group [18]. Our studies identified a new function of BRUCE in the ubiquitination and deubiquitination regulation of DNA damage repair and signaling. DNA damage induction activates DNA damage response for DNA repair and preservation of genome stability. We showed that a fraction of BRUCE protein is localized to the cell nucleus and even associated with chromatin. This nuclear pool of BRUCE regulates DNA repair and signaling. Our data demonstrated that BRUCE works together with a deubiquitinating (Dub) enzyme, the ubiquitin-specific peptidase 8 (USP8), to facilitate deubiquitination of the tumor suppressor and DNA damage response protein BRIT1/ MCPH1. This deubiquitination triggers the release of BRIT1 from the BRUCE-USP8-BRIT1 complex and consequently released BRIT acquires the ability to be recruited to sites of DNA damage by binding to phosphorylated H2AX (γ-H2AX). Thus, by regulating BRIT1 function, BRUCE contributes to subsequent recruitment of other essential DNA repair factors to damaged chromatin to accomplish DNA repair. Owing to the multiple critical roles that BRUCE plays in DNA repair and signaling, apoptosis, and cell division, BRUCE is essential for mouse embryonic development [13,19,20].

It has been demonstrated that elevated BRUCE expression is associated with treatment resistance, poor prognosis, or disease reoccurrence in prostate, ovarian, colon and melanoma cancers [21–24]. At the cellular level, higher levels of endogenous BRUCE correlate with better cell survival in response to chemotherapeutic drugs. Conversely, knockdown of BRUCE promotes apoptosis in human cancer cell lines [8,13,14,25,26]. Despite these studies, little is known with the implication of BRUCE in breast cancer. One study showed that knockdown of BRUCE in breast cancer cell lines ZR75.1 and MDA-MB-231 sensitizes them to apoptosis, but did not examine the effect of BRUCE knockdown on the cell sensitivity in response to chemotherapeutic agents [25]. Therefore, it remains to be investigated whether in BRUCE expression differs between normal and malignant primary breast cancer tissues and whether BRUCE levels contribute to breast cancer chemoresistance.

Our first objective was to use immunohistochemistry to compare protein levels of BRUCE in primary breast tumors and normal breast tissue. Our second objective was to determine if depletion of BRUCE, using gene silencing by lentiviral shRNA, can sensitize chemoresistant breast cancer cells to the cytotoxicity of several chemotherapeutic agents, including cisplatin and taxol. Using these approaches we found higher levels of BRUCE protein in tumor than in normal breast tissue. We also found that knockdown of BRUCE resulted in reduced cell viability in response to double agent treatment of cisplatin and taxol than when either drug was used alone. Our results suggest that the presence or absence of BRUCE expression in breast tumors can dictate the course of combination therapy with cisplatin and taxol. The future development of effective inhibitors of BRUCE could benefit patients with high levels of BRUCE expression and chemoresistance.

## Methods

### Cell lines and reagents

Breast cancer cell lines MDA-MB-231, MDA-MB-468 and MCF-7 were from American Type Culture Collection (ATCC) and grown in Dulbecco’s modified Eagle’s medium supplemented with 10% fetal bovine serum, and 1% penicillin/streptomycin. Cisplatin was purchased from Sigma-Aldrich. Taxol, staurosporine, and etoposide were a gift from Dr. Marshall Anderson. Human pLKO.1 lentiviral shRNA target gene set towards BRUCE was purchased from Open Biosystems. The five shRNAs targeting BRUCE, numbered as #7 to #11 in the manuscript correspond to the vendor’s catalog numbers of TRCN0000004157, TRCN0000004158, TRCN0000004159, TRCN0000004160, and TRCN0000004161, respectively. Lentiviral production was carried out according to manufacturer’s protocol.

### Lentivirus production and infection of breast cancer cell lines with lentiviral shRNA

Lentivirus containing shBRUCE was produced as followed: Phoenix cells were seeded at 7 × 10^5^ cells per 60-mm plate and the next day transfected with 1.0 μg of shBRUCE plasmid, 0.75 μg psPAX2, and 0.25 μg pMD2.G by Lipofectamine 2000 reagent (Invitrogen). The next day, the medium was replaced with new medium. After an additional 36 hours in culture, the medium containing the lentivirus and some Phoenix cells was collected. The Phoenix cells were removed by filtration with a 0.45-uM filter and the resulting fresh lentiviral solution was used directly to infect the breast cancer cell lines. Aliquots of the shRNA viral solution were used to infect breast cancer cell lines in culture for the time period of days as indicated in each experiment. The effect of BRUCE knockdown by the lentiviral shBRUCE was determined by immunoblotting detailed as follows.

### Immunoblotting

Cells were lysed post-infection in ice-cold cell lysis immunoprecipitation buffer [20 mM Tris-Cl (pH 7.5), 150 mM NaCl, 10% glycerol, 0.2% Nonidet P-40, and a protease inhibitor mixture (Roche Applied Science, Indianapolis, IN)]. Lysates were cleared of cell debris by centrifugation at 16,000×g at 4°C for 30 min. The supernatant was saved and aliquots corresponding to 20 μg total protein were used for SDS-PAGE/immunoblotting. Proteins were analyzed by SDS-PAGE and immunoblotted after transfer to nitrocellulose membranes (Bio-Rad Laboratories). Antibodies used include anti-actin and anti-FLAG M2 (Sigma), and anti-BRUCE (Calbiochem). An enhanced chemiluminescence (ECL) method (Pierce) was used for detection.

### Cell viability assay

Cells were seeded in 96-well plates at a density of 1000–3000 cells/well in 0.2 ml DMEM containing 10% FBS. Following overnight incubation medium was exchanged with 0.2 ml containing BRUCE lentivirus and/or various concentration of taxol, cisplatin, staurosporine, etoposide, or taxol/cisplatin. Cells were then allowed to incubate for 1–3 days. Finally, cell viability was accessed with an MTT reagent (Sigma-Aldrich), and by measuring the absorbance at 450 nm with a plate reader. Relative cell viability was obtained from the absorbance at 450 nm of the cells with drug treatment relative to that of cells without treatment. The same experiment was repeated with similar results.

### BRUCE immunohistochemical staining in breast tumors and expression scores

Breast tumor paraffin microarray slides (BioChain Institute, T8235721) were re-diagnosed by our pathologist (Jiang Wang). It contains 57 primary breast tumors with invasive ductal carcinoma being the major type and a few cases of invasive lobular carcinoma. It also contains 16 cases of normal breast tissue. BRUCE antibody (Bethyl Laboratories, A300-367A) was used and the antibody specificity to BRUCE was validated by the vendor. The antibody was used at 1:5000 dilution Epitope retrieval was with citrate buffer pH 6.0. Detection was by DAB staining using an immunohistochemistry VECTASTAIN ABC kit (Vector Laboratories, PK-6100) following manufacturer’s instruction. BRUCE expression level in breast tumor tissues was primarily differentiated by the number of positive cells as the staining varied little in intensity. Therefore, score 0 indicates less than 25% cells across the entire piece of tissue are positive for BRUCE staining; score 1, 2, and 3 indicates 25%–50%, 50%–75%, and higher than 75% of cells positive for BRUCE, respectively.

### Statistical analysis

The average score values of normal vs tumor tissues were compared and analyzed by Student’s *t*-test (unpaired), results with standard error of the mean (SEM), *P*<0.05 was considered significant (one-tailed test).

## Results and Discussion

### BRUCE protein level is significantly elevated in human primary breast tumor tissues

**Figure 1:**
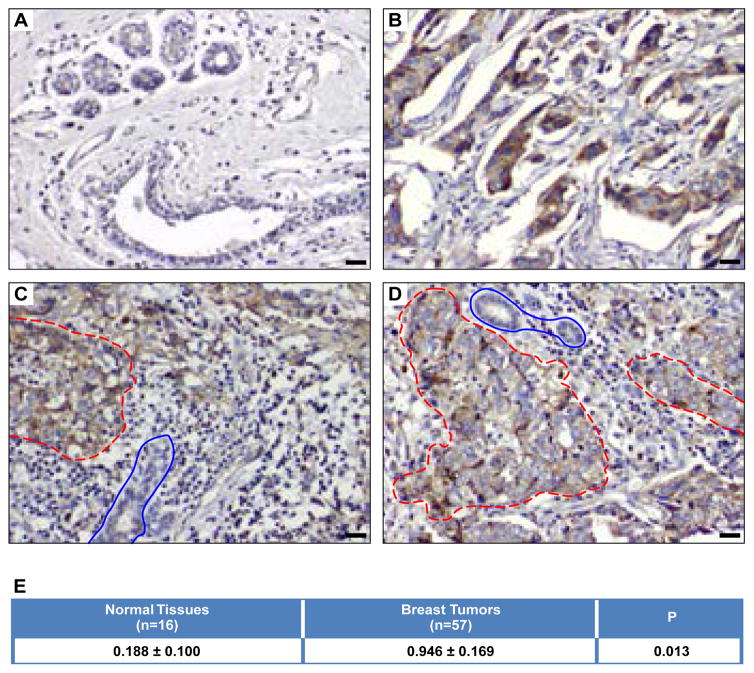
The level of BRUCE protein is upregulated in breast tumor tissues IHC of BRUCE on normal breast tissue (**A**) and primary breast tumors (**B–D**) on paraffin array slides with tumor tissue outlined in red circles and adjacent normal tissues in blue circles; scale bar: 20 mircometer. Statistical analysis of BRUCE IHC staining (**E**) was based on clinic scoring criteria described in the Methods. The values represent average scores of normal vs tumor tissues. Variation represents standard error of the mean (SEM). Student’s *t*-test, *P*=0.013; (*p*<0.05 was regarded significant; one-tailed test).

We first examined whether the levels of BRUCE protein differ in normal and breast tumor tissues. IHC of BRUCE was conducted in a breast tumor tissue array. It consists of 16 normal tissues and 57 breast tumors with invasive ductal carcinomas being the major type. Results of BRUCE IHC showed that the levels of BRUCE staining and the percentage of positivity were much higher in breast tumors than in normal breast tissues (Figure 1A and 1B). Moreover, in any given tumor sample on this array, BRUCE expression was significantly elevated in malignant cells over adjacent normal cells (Figure 1B–1D). Furthermore, BRUCE staining was mainly present in the cytoplasm of tumor cells (Figure 1B–1D), which was in agreement with the previous report that BRUCE localization is mainly found in the cytoplasm of a cell [7].

Based upon these observations, each tissue on the array was scored for BRUCE IHC by our pathologist based on the clinic scoring criteria described in the Methods. Statistical analysis of the scores showed that the mean percentage of BRUCE expression was significantly elevated in breast tumors compared to normal breast tissue (Figure 1E, *p*=0.013; *p*<0.05 was regarded significant). It is noted that this study cannot conclude the correlation of BRUCE expression levels with different subtypes of breast tumors, for instance, estrogen and progesterone receptors and Her2 expression. Nonetheless, the elevated BRUCE levels in the overall breast cancer samples provide the advantage of potentially using BRUCE as a general target of therapy for a broad spectrum of breast cancers.

### Sensitivity of breast cancer cell lines to chemotherapeutic agents

**Figure 2:**
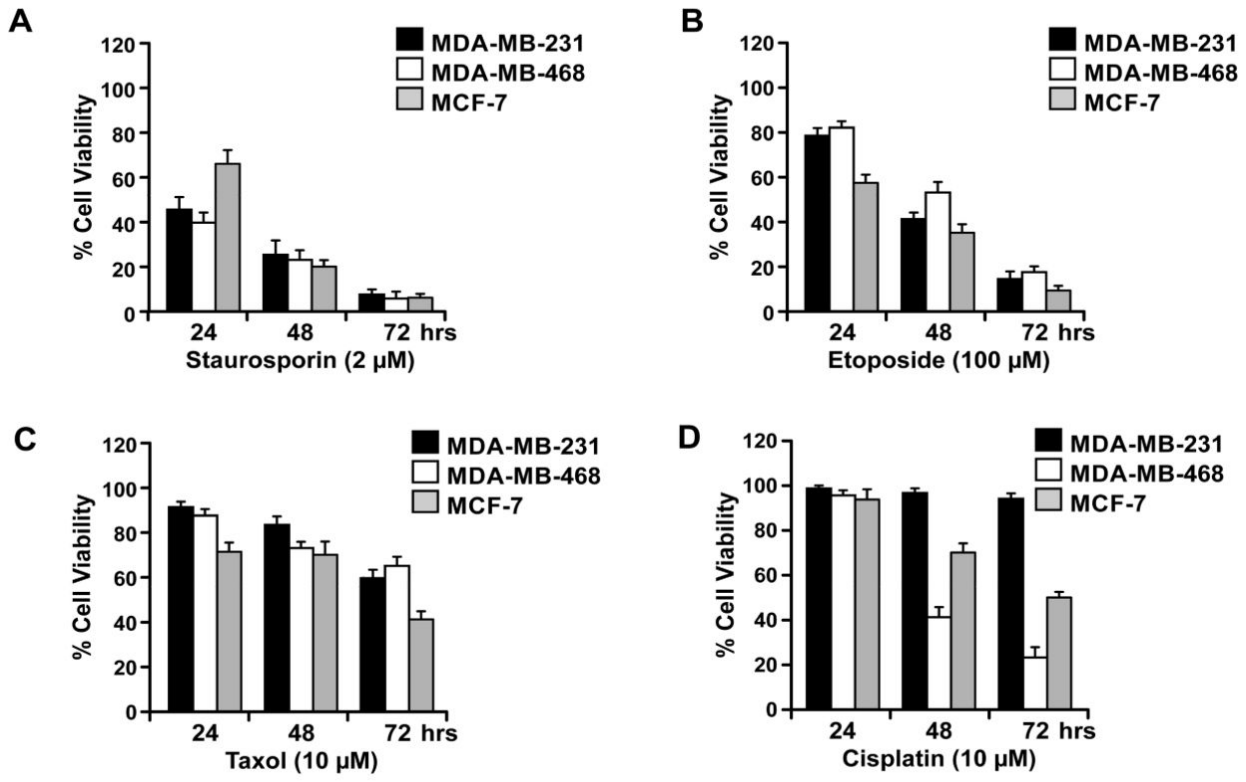
Breast cancer cell sensitivity to chemotherapeutic drugs Breast cancer MDA-MB-231, MDA-MB-468, and MCF-7 cell lines were treated with staurosporine at 2 μM (**A**), etoposide at 100 μM (**B**), Taxol at 10 μM (**C**), and cisplatin at 10 μM (**D**). Cell viability was assessed by MTT assay at 24, 48, and 72 hrs post drug treatment. Result of three independent experiments +/− SEM.

The elevated expression of BRUCE in human breast tumors suggests that BRUCE could contribute to the chemoresistance in breast cancer patients. To explore this possibility, we investigated if reducing the protein levels of BRUCE sensitizes breast cancer cell lines to chemotherapeutic agents. Based on the three functions of BRUCE in DNA damage and repair, cytokinesis, and apoptosis suppression, the DNA cross-linker cisplatin and the mitotic inhibitor taxol were selected for the study in breast cancer cell lines of MDA-MB-231, MDA-MB-468 and MCF-7. In addition, two other commonly used chemotherapeutic drugs, the protein kinase inhibitor staurosporine which triggers potent apoptosis, and the topoisomerase inhibitor etoposide, were also examined. Cell viability was determined by MTT assay post 24, 48, and 72 hrs of treatment with each drug. Exposure of these cells to staurosporine, etoposide and taxol resulted in significant and time dependent reduction of cell viability in all three cell lines (Figure 2A–2C, respectively). In contrast, cells exposed to cisplatin displayed differential cytotoxicity, with stronger toxicity on MDA-MB-468 and MCF7 and weaker on MDA-MB-231 cells, as by 72 hrs of exposure the latter still remained nearly 100% viable whereas the former two cell lines already lost more than 50% viability (Figure 2D). These results indicate that all three cell lines are sensitive to staurosporine, etoposide and taxol and that MDA-MB-231 cell are more resistant to cisplatin under the current experimental conditions. These results prompted us to further examine whether the cisplatin resistance exhibited by MDA-MB-231 cells can be alleviated by lowering the levels of BRUCE expression in the following studies.

### Reduction in BRUCE protein levels via shRNA mediated lentiviral knockdown

**Figure 3:**
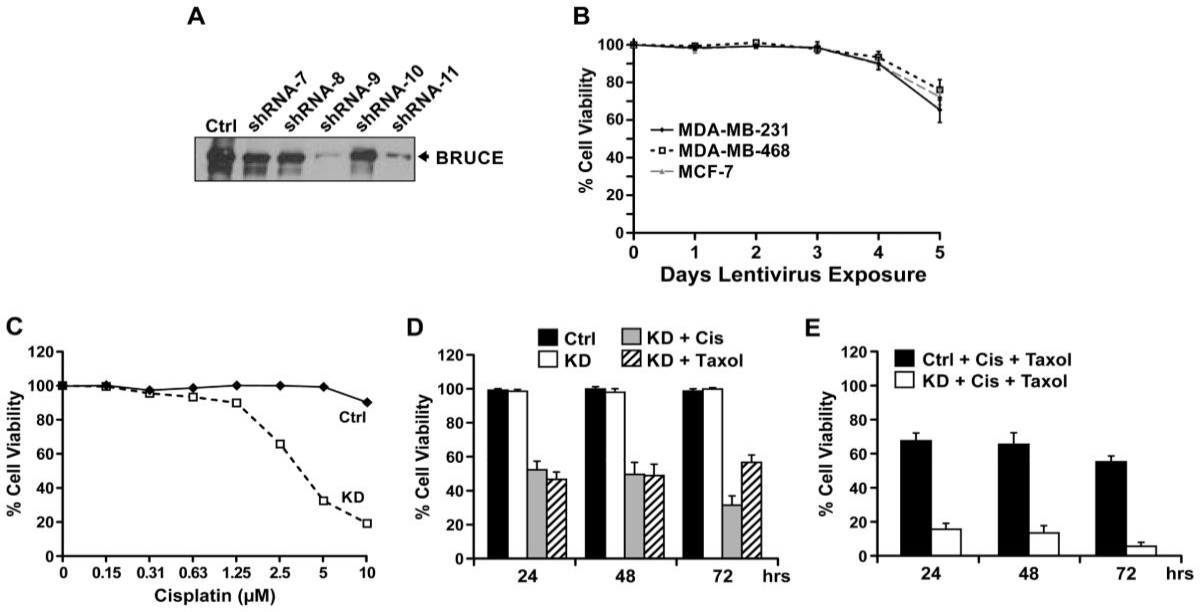
BRUCE knockdown sensitizes resistant MDA-MB-231 cells to cisplatin and taxol-induced cell death (**A**) Western blot of BRUCE protein levels preand post-lentiviral knockdown for 48 hrs in MDA-MB-231 cells exposed to lentivirus expressing five BRUCE-targeted shRNA (#7 – #11) and a scramble control (ctrl). (**B**) MTT assay of MDA-MB-231, MDA-MB-468, and MCF-7 cell lines following 1–5 days of lentiviral exposure (shRNA #9). Result of three independent experiments +/− SEM. (**C**) MDA-MB-231 cells treated with control or shBRUCE lentivirus for two days were exposed to increasing concentrations of cisplatin (0–10 μM) for three days and cell viability was measured by MTT assay. (**D**) MDA-MB-231 cells were exposed to shBRUCE lentivirus for two days followed by treatment with cisplatin (5 μM) or taxol (0.65 μM) for the indicated time points. Following treatment, the MTT assay for cell viability was performed. (**E**) MDA-MB-231 cells exposed to shBRUCE lentivirus for two days were co-exposed to cisplatin (2.5 μM) and taxol (0.65 μM) for the indicated time points. Following treatment the MTT assay for cell viability was performed. Result of three independent experiments +/− SEM.

With the aim to determine if knockdown of BRUCE sensitizes resistant breast cancer cells to chemotherapeutic drugs, we screened five different BRUCE-targeted lentiviral shRNAs for their effect on BRUCE knockdown in the three cell lines. Western blot results demonstrated that shRNAs #9 and #11 were most effective for knockdown of BRUCE after 2 days of lentiviral shRNA infection in all three cell lines as exemplified in MDA-MB-231 cells (Figure 3A). Therefore, shRNA #9 was used in the following experiment to assess the cell viability over a course of 5 days of lentivirus-mediated knockdown of BRUCE. Results of MTT viability assay demonstrated that all three cell lines still exhibited 100% viability post 2 days of shRNA treatment, however, BRUCE was already depleted at this time. The cell viability continued to remain at 100% on day 3, slightly dropped on day 4, and further reduced on day 5 (Figure 3B). These results indicate that loss of cell viability does not occur simultaneously with loss of BRUCE expression, but ensures two days afterwards. Thus, lentiviral shRNA treatment for 2 days, during which BRUCE knockdown has been achieved and cell viability is still well preserved, was used in the following experiments to test the effect of BRUCE depletion on cell sensitivity to chemotherapeutic drugs.

### BRUCE knockdown enhances chemotherapeutic drug triggered cell death

BRUCE knockdown reduces the DNA repair capacity [18]. We examined if BRUCE knockdown enhances the responsiveness of chemoresistant MDA-MB-231 cells to the DNA damaging agent cisplatin. Cells were infected with the BRUCE-targeted shRNA lentivirus followed by exposure to increasing concentrations of cisplatin (0–10 μM). The MTT results showed that cisplatin treatment of BRUCE knockdown cells resulted in a reduction of cell viability in a dose dependent manner (Figure 3C). Specifically, only 19% BRUCE-knockdown cells remained viable after incubation with 10 μM cisplatin for 3 days, whereas the viability of control cells infected with scramble shRNA was well preserved (Figure 3C). These results demonstrate that knockdown of BRUCE sensitizes the resistant MDA-MB-231 cells to cisplatin. Since cisplatin treatment alone at 5 μM resulted in a moderate reduction in cell viability (Figure 3C), this dose was used in conjunction with BRUCE knockdown to assess if BRUCE depletion could enhance cell sensitivity to cisplatin. To do this, MDA-MB-231 cells were transduced with BRUCE-targeted shRNA lentivirus followed by exposure to cisplatin for 24–72 hrs. The results showed 100% viability in the control or in BRUCE knockdown cells at each time point (Figure 3D, solid and open bars). In contrast, cells co-treated with BRUCE shRNA and 5 μM cisplatin displayed significant reduction in cell viability at 24 and 48 hrs (54%) and further reduction at 72 hrs (30%) (Figure 3D, gray bars). This indicated that depletion of BRUCE can sensitize the cells to cisplatin.

In addition to promoting DNA damage response and repair, BRUCE promotes cytokinesis and cell division. As a result, depletion of BRUCE increases the incidence of abortive cell division, leading to cell death [12]. Prompted by the cytokinesis function of BRUCE, we investigated if knockdown of BRUCE sensitizes cells to cell division blocker taxol. The cell viability results showed that knockdown of BRUCE indeed made taxol more cytotoxic to the cells, even at a low concentration of 0.65 μM (Figure 3D, dash bars).

It has been demonstrated in clinical trials that combination of different chemotherapeutic drugs often enhances efficacy over single-agent chemotherapy in breast and other cancers [27–29]. Therefore, we assessed whether depletion of BRUCE could more significantly increase cell sensitivity to cisplatin in combination with taxol. Interestingly, concurrent exposure of BRUCE-knocked down MDA-MB-231 cells to both cisplatin (2.5 μM) and taxol (0.65 μM) elicited a more significantly reduced cell viability at all the time points examined (Figure 3E). It should be noted that knockdown of XIAP (another IAP family member) failed to sensitize these cells to cisplatin and/or taxol (data not shown). Together, these results indicate that reducing BRUCE expression significantly enhances the cytotoxicity of combined treatment with cisplatin and taxol in otherwise chemoresistant MDA-MB-231 cells.

**Figure 4:**
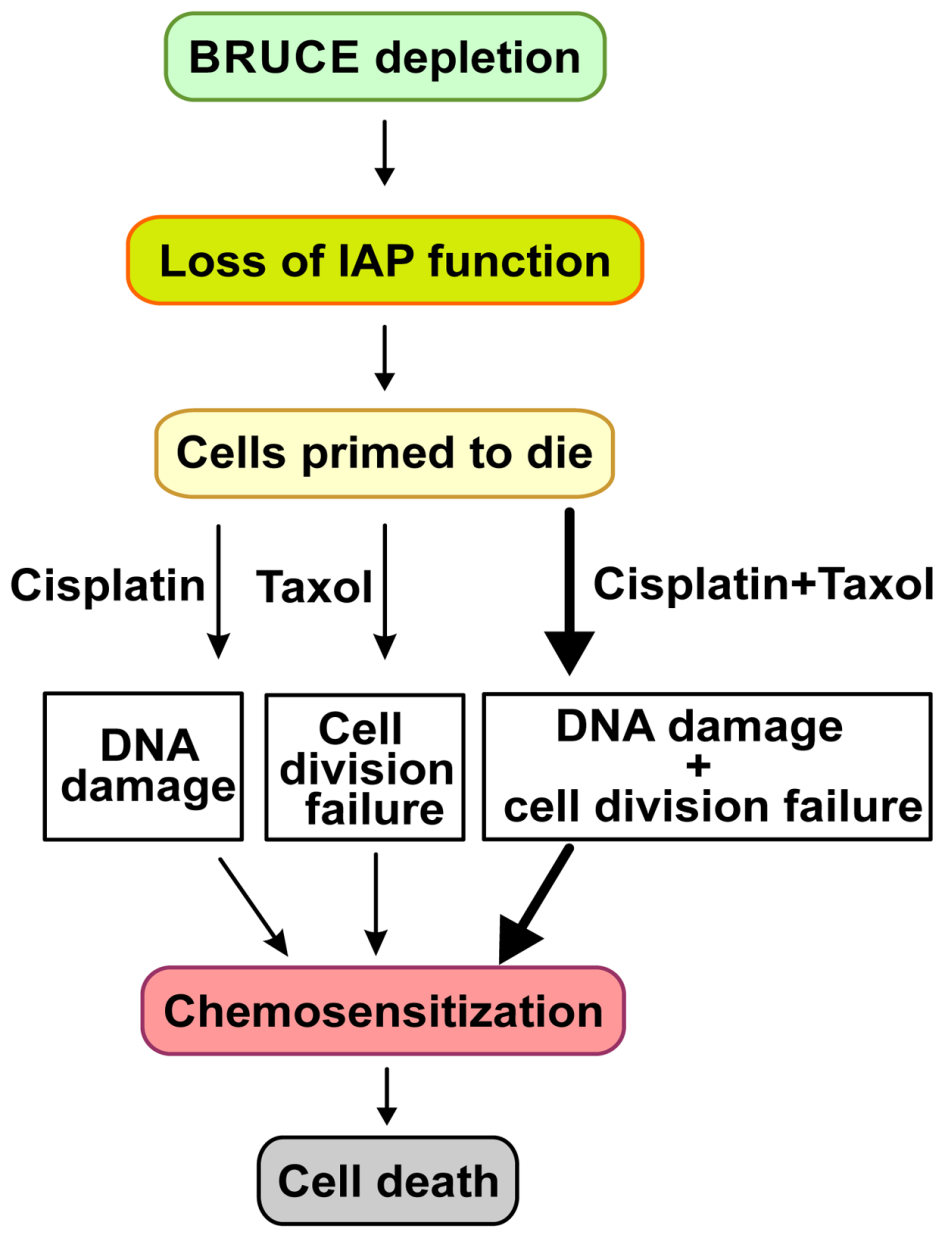
A working model for how BRUCE depletion sensitizes breast cancer cells to chemotherapy Contribution of BRUCE to the chemoresistance in breast cancers comes from BRUCE functions in anti-apoptosis, pro-cytokinesis, and DNA repair. Depletion of BRUCE results in loss of its IAP function and therefore cells are primed to the cytotoxicity of chemotherapy. BRUCE is also required for cell division and DNA damage repair. When BRUCE depletion is coupled to the cell division blocker taxol or DNA damage inducer cisplatin, the overall degree of cell viability reduces (thin arrows). Moreover, once the two pathways of cell division and DNA repair are hit simultaneously by the dual drug treatment, the chemotherapeutic treatments elicit a more profound impairment of cell viability in the already primed cells depleted of BRUCE (thick arrow).

Based on the data presented in this study and the known cellular functions of BRUCE in DNA damage and repair, anti-apoptosis and pro-cytokinesis, it is conceivable that the contribution of BRUCE depletion to chemo-sensitization of breast cancer cells comes from at least lacking these three functions of BRUCE, which is as summarized in Figure 4. Depletion of BRUCE results in loss of its IAP function and therefore makes the cells primed to the cytotoxicity of chemotherapeutic agents. When cisplatin and taxol are delivered to the primed cells, they hit two essential cellular functions of BRUCE in DNA repair and cytokinesis, respectively and results in more profound cell death. Therefore, the synergistically enhanced cellular toxicity by dual treatment with cisplatin and taxol in BRUCE-depleted cells is likely achieved by simultaneous inactivation of multiple cellular processes in which BRUCE plays critical roles (Figure 4).

## Conclusions

This BRUCE study demonstrates a significant elevation of BRUCE protein levels in primary breast tumors. It also indicates that reducing BRUCE expression in breast cancer cells promotes cell killing by dual treatment with cisplatin and taxol. Hence, our findings suggest that reducing BRUCE protein levels could sensitize chemotherapy-refractory breast cancer patients to two-agent treatment of cisplatin and taxol. It is therefore tempting to propose a new course of combinational treatment consisting of cisplatin and taxol together with inhibition of BRUCE for resistant breast cancer patients.

## Abbreviations

IAP: Inhibitor of Apoptosis Protein
Birc6: Baculoviral IAP Repeat Containing 6
MTT: 3-(4,5-Dimethylthiazol-2-Yl)-2,5-Diphenyltetrazolium Bromide

## References

[1] Holohan C, Van Schaeybroeck S, Longley DB, Johnston PG (2013). Cancer drug resistance: an evolving paradigm. Nat Rev Cancer.

[2] Weigelt B, Peterse JL, van’t Veer LJ (2005). Breast cancer metastasis: markers and models. Nat Rev Cancer.

[3] Gascoigne KE, Taylor SS (2009). How do anti-mitotic drugs kill cancer cells?. J Cell Sci.

[4] Kelland L (2007). The resurgence of platinum-based cancer chemotherapy. Nat Rev Cancer.

[5] Mann J1 (2002). Natural products in cancer chemotherapy: past, present and future. Nat Rev Cancer.

[6] Vargo-Gogola T, Rosen JM (2007). Modelling breast cancer: one size does not fit all. Nat Rev Cancer.

[7] Hauser HP, Bardroff M, Pyrowolakis G, Jentsch S (1998). A giant ubiquitin-conjugating enzyme related to IAP apoptosis inhibitors. J Cell Biol.

[8] Chen Z, Naito M, Hori S, Mashima T, Yamori T (1999). A human IAP-family gene, apollon, expressed in human brain cancer cells. Biochem Biophys Res Commun.

[9] Salvesen GS, Duckett CS (2002). IAP proteins: blocking the road to death’s door. Nat Rev Mol Cell Biol.

[10] Verhagen AM, Coulson EJ, Vaux DL (2001). Inhibitor of apoptosis proteins and their relatives: IAPs and other BIRPs. Genome Biol.

[11] Bartke T, Pohl C, Pyrowolakis G, Jentsch S (2004). Dual role of BRUCE as an antiapoptotic IAP and a chimeric E2/E3 ubiquitin ligase. Mol Cell.

[12] Pohl C, Jentsch S (2008). Final stages of cytokinesis and midbody ring formation are controlled by BRUCE. Cell.

[13] Ren J, Shi M, Liu R, Yang QH, Johnson T (2005). The Birc6 (Bruce) gene regulates p53 and the mitochondrial pathway of apoptosis and is essential for mouse embryonic development. Proc Natl Acad Sci U S A.

[14] Hao Y, Sekine K, Kawabata A, Nakamura H, Ishioka T (2004). Apollon ubiquitinates SMAC and caspase-9, and has an essential cytoprotection function. Nat Cell Biol.

[15] Qiu XB, Goldberg AL (2005). The membrane-associated inhibitor of apoptosis protein, BRUCE/Apollon, antagonizes both the precursor and mature forms of Smac and caspase-9. J Biol Chem.

[16] Du C, Fang M, Li Y, Li L, Wang X (2000). Smac, a mitochondrial protein that promotes cytochrome c-dependent caspase activation by eliminating IAP inhibition. Cell.

[17] Sekine K, Hao Y, Suzuki Y, Takahashi R, Tsuruo T (2005). HtrA2 cleaves Apollon and induces cell death by IAP-binding motif in Apollon-deficient cells. Biochem Biophys Res Commun.

[18] Ge C, Che L, Ren J2, Pandita RK3, Lu J (2015). BRUCE regulates DNA double-strand break response by promoting USP8 deubiquitination of BRIT1. Proc Natl Acad Sci U S A.

[19] Lotz K, Pyrowolakis G, Jentsch S (2004). BRUCE, a Giant E2/E3 Ubiquitin Ligase and Inhibitor of Apoptosis Protein of the trans-Golgi Network, Is Required for Normal Placenta Development and Mouse Survival. Mol Cell Biol.

[20] Hitz C, Vogt-Weisenhorn D, Ruiz P, Wurst W, Floss T (2005). Progressive loss of the spongiotrophoblast layer of Birc6/Bruce mutants results in embryonic lethality. Genesis.

[21] Wang L, Chen YJ, Hou J, Wang YY, Tang WQ (2014). Expression and clinical significance of BIRC6 in human epithelial ovarian cancer. Tumour Biol.

[22] Luk SU, Xue H, Cheng H, Lin D, Gout PW (2014). The BIRC6 gene as a novel target for therapy of prostate cancer: dual targeting of inhibitors of apoptosis. Oncotarget.

[23] Van Houdt WJ, Emmink BL, Pham TV, Piersma SR, Verheem A (2011). Comparative proteomics of colon cancer stem cells and differentiated tumor cells identifies BIRC6 as a potential therapeutic target. Mol Cell Proteomics.

[24] Tassi E, Zanon M, Vegetti C, Molla A, Bersani I (2012). Role of Apollon in human melanoma resistance to antitumor agents that activate the intrinsic or the extrinsic apoptosis pathways. Clin Cancer Res.

[25] Lopergolo A, Pennati M, Gandellini P, Orlotti NI, Poma P (2009). Apollon gene silencing induces apoptosis in breast cancer cells through p53 stabilisation and caspase-3 activation. Br J Cancer.

[26] Qiu XB, Markant SL, Yuan J, Goldberg AL (2004). Nrdp1-mediated degradation of the gigantic IAP, BRUCE, is a novel pathway for triggering apoptosis. EMBO J.

[27] Robertson JF, Dixon JM, Sibbering DM, Jahan A, Ellis IO (2013). A randomized trial to assess the biological activity of short-term (pre-surgical) fulvestrant 500 mg plus anastrozole versus fulvestrant 500 mg alone or anastrozole alone on primary breast cancer. Breast Cancer Res.

[28] Shaughnessy JN, Meena RA, Dunlap NE, Jain D, Riley EC (2014). Efficacy of Concurrent Chemoradiotherapy for Patients With Locally Recurrent or Advanced Inoperable Breast Cancer. Clin Breast Cancer.

[29] Lesnock JL, Darcy KM, Tian C, Deloia JA, Thrall MM (2013). BRCA1 expression and improved survival in ovarian cancer patients treated with intraperitoneal cisplatin and paclitaxel: a Gynecologic Oncology Group Study. Br J Cancer.

